# AICAR Protects Vascular Endothelial Cells from Oxidative Injury Induced by the Long-Term Palmitate Excess

**DOI:** 10.3390/ijms23010211

**Published:** 2021-12-25

**Authors:** Mikhail V. Samsonov, Nikita V. Podkuychenko, Asker Y. Khapchaev, Eugene E. Efremov, Elena V. Yanushevskaya, Tatiana N. Vlasik, Vadim Z. Lankin, Iurii S. Stafeev, Maxim V. Skulachev, Marina V. Shestakova, Alexander V. Vorotnikov, Vladimir P. Shirinsky

**Affiliations:** 1 National Medical Research Center for Cardiology, 121552 Moscow, Russia; mvs.laba@gmail.com (M.V.S.); nra.fox@gmail.com (N.V.P.); askerkhapcha@gmail.com (A.Y.K.); efremoveugene@rambler.ru (E.E.E.); yanushevskaya@yandex.ru (E.V.Y.); tanya.vlasik@gmail.com (T.N.V.); lankin0309@mail.ru (V.Z.L.); yuristafeev@gmail.com (I.S.S.); 2 Belozersky Institute of Physico-Chemical Biology, M. V. Lomonosov Moscow State University, 119234 Moscow, Russia; max@mitotech.ru; 3 Diabetes Institute, Endocrinology Research Center, 117036 Moscow, Russia; shestakova.mv@gmail.com

**Keywords:** HUVEC, endothelial barrier, hyperlipidemia, insulin signaling, palmitate, NO, ROS, malondialdehyde

## Abstract

Hyperlipidemia manifested by high blood levels of free fatty acids (FFA) and lipoprotein triglycerides is critical for the progression of type 2 diabetes (T2D) and its cardiovascular complications via vascular endothelial dysfunction. However, attempts to assess high FFA effects in endothelial culture often result in early cell apoptosis that poorly recapitulates a much slower pace of vascular deterioration in vivo and does not provide for the longer-term studies of endothelial lipotoxicity in vitro. Here, we report that palmitate (PA), a typical FFA, does not impair, by itself, endothelial barrier and insulin signaling in human umbilical vein endothelial cells (HUVEC), but increases NO release, reactive oxygen species (ROS) generation, and protein labeling by malondialdehyde (MDA) hallmarking oxidative stress and increased lipid peroxidation. This PA-induced stress eventually resulted in the loss of cell viability coincident with loss of insulin signaling. Supplementation with 5-aminoimidazole-4-carboxamide-riboside (AICAR) increased endothelial AMP-activated protein kinase (AMPK) activity, supported insulin signaling, and prevented the PA-induced increases in NO, ROS, and MDA, thus allowing to maintain HUVEC viability and barrier, and providing the means to study the long-term effects of high FFA levels in endothelial cultures. An upgraded cell-based model reproduces FFA-induced insulin resistance by demonstrating decreased NO production by vascular endothelium.

## 1. Introduction

Dyslipidemia, manifested by elevated levels of plasma triglycerides and FFA, is tightly associated with obesity, peripheral insulin resistance, and increased risk of T2D [1,2]. Normally, insulin activates endothelial NO-synthase (eNOS) in endothelial cells to promote vasodilation, increase the blood flow and insulin delivery to underlying tissues [3]. In dyslipidemia, vascular endothelium is the primary target of blood FFA. Whether or not liberated from albumin as an FFA carrier, or from lipoprotein particles by endothelium-associated lipoprotein lipase, the FFA levels are chronically increased at the endothelial interface, leading to its dysfunction and insulin resistance [4,5,6]. In response to increased FFA levels, endothelial cells develop oxidative stress, upregulate endothelin-1 and lower NO production, downregulate insulin signaling, thus shifting the vasculature into hypertensive mode. These events are not only thought to link T2D to its cardiovascular complications [7,8,9], but are even considered as the primary cause of T2D [4,10].

Molecular mechanisms of vascular endothelial dysfunction in obesity and dyslipidemia are not fully explored, largely because of restricted access to this tissue in vivo, and the absence of appropriate cell models in vitro. Experimental models of hyperlipidemia commonly use palmitic acid as a typical FFA and HUVEC [11,12,13,14,15,16,17,18], producing results compatible with other endothelial cell models [19,20,21]. They linked FFA-induced oxidative stress to increased ROS in the primary form of O_2_-superoxide anion radical, suggesting their mitochondrial origin. MDA is an established cardiovascular marker of increased oxidative stress and lipid peroxidation [22]. It impairs insulin signaling and barrier function in endothelial cells [23,24], however, whether it is involved in responses to increased FFA is unknown. Neither is known whether elevated FFA and associated oxidative stress alter the endothelial barrier, the key defense of underlying cells.

The plasma concentrations of albumin-bound FFA range from 0.1 mM to 0.7 mM, subject to their diurnal fluctuations and variable composition with respect to molecular mass [1]. Vast evidence is now available that non-esterified FFA, usually typified by PA, abruptly triggers endothelial cell apoptosis in vitro [11,12,13,14,17,18,19,20,21,25]. HUVEC could not be maintained longer than 2 days even at low PA (or other FFA) concentrations that are compatible with their normal levels in human blood. While these studies appear to converge on the conclusion that FFA-induced apoptosis is the endmost fate of vascular endothelium in T2D, substantial apoptosis of vascular endothelium in vivo is often attributed only to the end-stage T2D manifested by impaired limb angiogenesis and ulceration, associated with inflammation [26,27], atherosclerosis [28], or other diseases [27]. This discrepancy suggests that additional supplements may improve the viability of endothelial cells and confer them appropriate reactivity to FFA in vitro.

Early studies have demonstrated that carnitine, an essential component of carnitine-palmitoyl transferase that delivers the long fatty acid acyl moieties to mitochondria for oxidation, is easily lost by cultured endothelial cells and could be readily accumulated from outside leading to enhanced fatty acid oxidation (FAO) [29,30,31]. When combined with AICAR, the AMPK activator, carnitine counteracted the high glucose-induced HUVEC apoptosis [32] and inflammatory response [33]. Whereas these studies aimed to demonstrate the principal ability of endothelial cells to shift their carbohydrate metabolism to FAO with a critical contribution of AMPK, they did not pursue the effects of carnitine and AICAR on long-term viability and sustainability of endothelial cells to high FFA levels typical for dyslipidemia.

Here, we extended these studies to demonstrate that supplemental carnitine and AICAR prevent PA-induced early cell death of HUVEC cultured in a defined medium, and sustain endothelial viability and barrier for at least two weeks. Optimized culture conditions allow long-term studies of endothelial reactivity in obesity/diabetes/cardiovascular pathologies. In this culture system, acute oxidative cell injury by excessive PA is diminished whereas the interference of PA with insulin stimulation of NO generation by endothelial cells persists. Moreover, our results further strengthen the potential of endothelial AMPK, activated by AICAR, as a promising therapeutic target in metabolic dysfunctions.

## 2. Results

Numerous studies reported a significant rate of HUVEC apoptosis over 48–72 h of cell treatment with PA even in the media specifically designed for endothelial cell growth, such as EGM (Lonza) or mv-EGM (Cell Applications). Supplementation of the cell media with carnitine and AICAR prevented early signs of PA-induced apoptosis [34], but their long-term effects on HUVEC viability have not been evaluated. Therefore, we designed the experiments first to explore the effects of AICAR/carnitine on the viability of vascular endothelium continuously treated by PA for a long time, and then to assess the principal markers of endothelial function, i.e., the barrier integrity, responses to insulin, and NO release.

### 2.1. AICAR Rescues HUVEC Viability in the Presence of Palmitate

**Figure 1:**
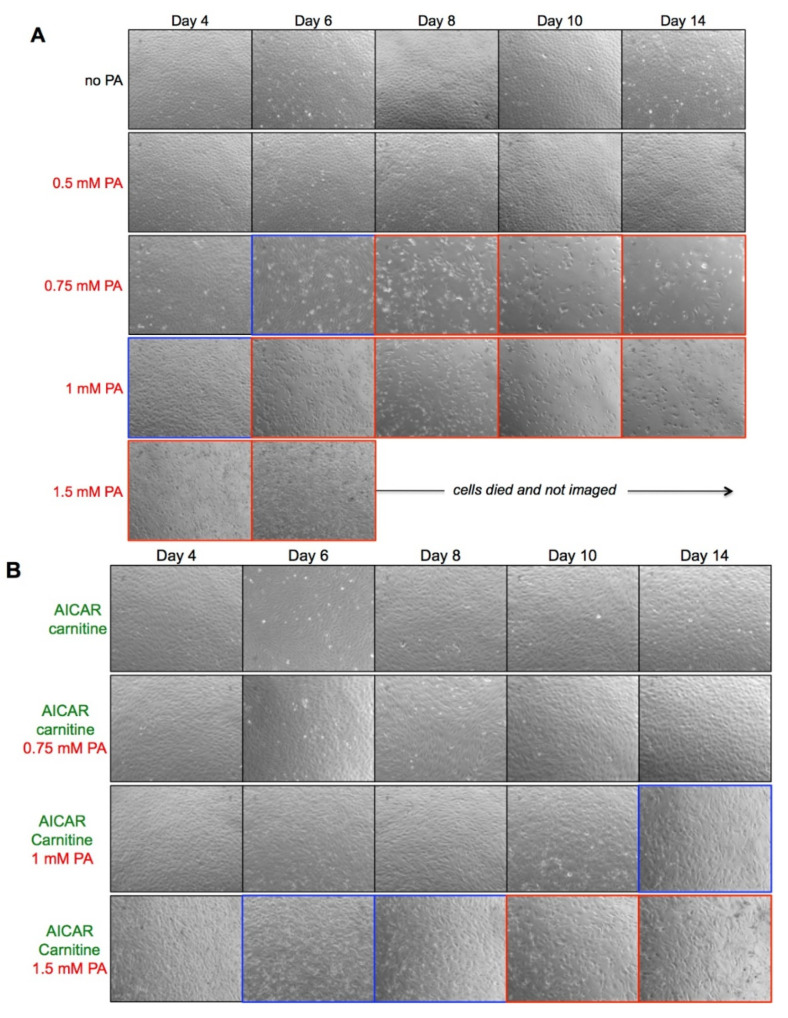
HUVEC viability is decreased by palmitate and rescued by AICAR/carnitine. The cells were cultured in EGM with different PA concentrations in absence (**A**), or presence of 40 μM carnitine and 1 mM AICAR (**B**) as indicated on the left. The medium was exchanged daily and HUVEC monolayers were examined by phase-contrast microscopy at the time points indicated on the top. Shown are the representative 10× images from 2 independent experiments. Blue frames mark the onset of apparent changes in cell monolayers, whereas the red frames denote clearly impaired cell monolayers. Full size annotated images corresponding to HUVEC + 0.75 mM PA, days 4–14 and to HUVEC + 0.75 mM PA, 1 mM AICAR/carnitine, days 4–14 could be found in Supplementary Materials. Other images are available on request.

We treated HUVEC monolayers with different concentrations of albumin-conjugated PA in the absence or presence of carnitine and AICAR (Figure 1). The cell viability was directly assessed by light microscopy. The chosen PA concentrations roughly correspond to physiological (0.5 mM), pre- (0.75 mM), or diabetic (≥1 mM) FFA levels in the blood plasma. Low PA concentrations (up to 0.5 mM) were well tolerated and the cell cultures were similar to the untreated by PA and maintained classic cobblestone appearance over 2 weeks. PA distorted the monolayers dose and time dependently; the onset of critical changes occurred on days 6 and 7 in 0.75 mM PA (indicated in blue in Figure 1A). Subsequent rarefaction of the monolayers indicating the cell loss and reduced viability occurred at day 8 in 0.75 mM PA, day 6 in 1 mM PA, and day 2 in 1.5 mM PA as indicated by red frames in Figure 1A. The effects of 0.75–0.8 mM PA were further explored as it roughly translates to the threshold fasting plasma FFA levels in individuals with obesity and/or T2D [1,35,36,37].

Supplementation of the cell medium with carnitine and AICAR rescued cell viability and allowed maintaining HUVEC monolayers for at least 2 weeks in the continuous presence of 0.75 mM PA (Figure 1B). In 1 mM PA the monolayers demonstrated altered morphology and gaps between cells after 14 days of culture. In the presence of 1.5 mM PA, the monolayers were apparently uniform till day 4 and then started to disperse clearly showing gaps and decreased number of cells after 10 days. Irrespective of PA presence, AICAR always altered the cobblestone cell morphology into an elongated spindle-like phenotype, which was apparently augmented by increasing PA concentrations. In the absence of AICAR, carnitine alone was unable to rescue the cell viability (not shown).

Thus, PA dramatically reduced HUVEC viability in a time- and dose-dependent manner, which was rescued by carnitine/AICAR. In the next series of experiments, we chose 0.75 mM PA to monitor permeability of HUVEC monolayers in the presence of carnitine/AICAR and to probe the molecular events underlying PA action.

### 2.2. AICAR Maintains the HUVEC Barrier in the Presence of Palmitate

**Figure 2:**
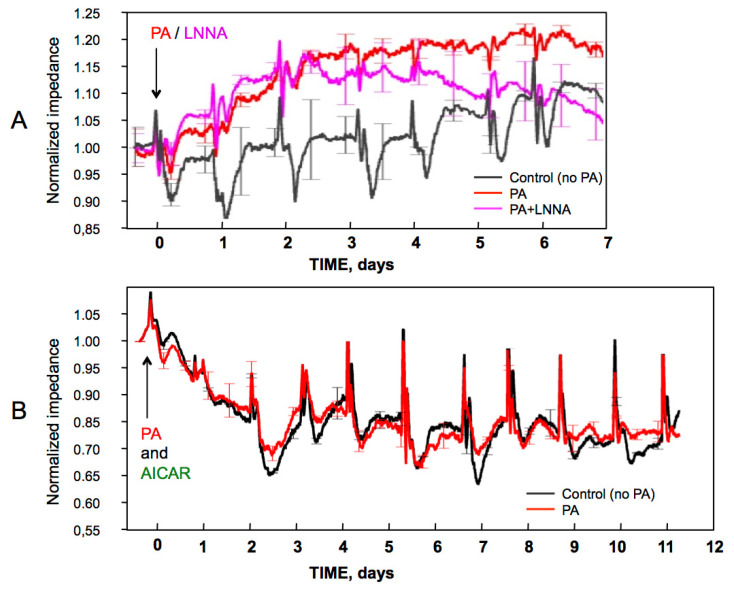
Effects of palmitate and/or AICAR/carnitine on transendothelial resistance (TER) of HUVEC monolayers. (**A**) PA increased TER of HUVEC monolayers in eNOS-dependent manner in the absence of AICAR. (**B**) AICAR negated the stimulatory effect of PA and moderately decreased TER, but the barrier was stably maintained at ~12 kOhm. PA, LNNA, or AICAR were applied at zero time as indicated by arrows. Cell media contained 40 μM carnitine and were exchanged daily. Representative results of three independent assays are shown; bars indicate variability of triplicates.

HUVEC monolayer permeability was measured by transendothelial resistance (TER). TER tracings were digitally normalized to initial TER values of 12–18 kOhm that had been established to validate the monolayer integrity before PA addition. Figure 2 shows representative TER tracings either for 6 days when the cells were viable and maintained monolayers in PA with no AICAR, or for 11 days in the presence of AICAR, the conditions when monolayers were stable for at least 2 weeks (c.f., Figure 1).

In the absence of AICAR, TER of the control cells steadily increased (Figure 2A, the black tracing), reflecting the tightening of the barrier. The addition of PA produced an even faster increase in TER by about 20% (Figure 2A, the red tracing). While this increase was initially insensitive to LNNA, which selectively inhibits eNOS/nNOS but not iNOS [38], its subsequent maintenance depended on eNOS activity as it was inhibited by LNNA (Figure 2A, the magenta tracing). LNNA had no effect on the control TER in the absence of PA (not shown). In the presence of AICAR, TER of both control and PA-treated cells became similar and lower than in the absence of AICAR. After the initial 3-day decline, the monolayer impedance plateaued at ~12 kOhm indicating sufficient barrier integrity. The PA-induced increase in TER was not reproduced in the presence of AICAR.

### 2.3. AMPK Activity Is Increased in AICAR-Treated HUVEC

**Figure 3:**
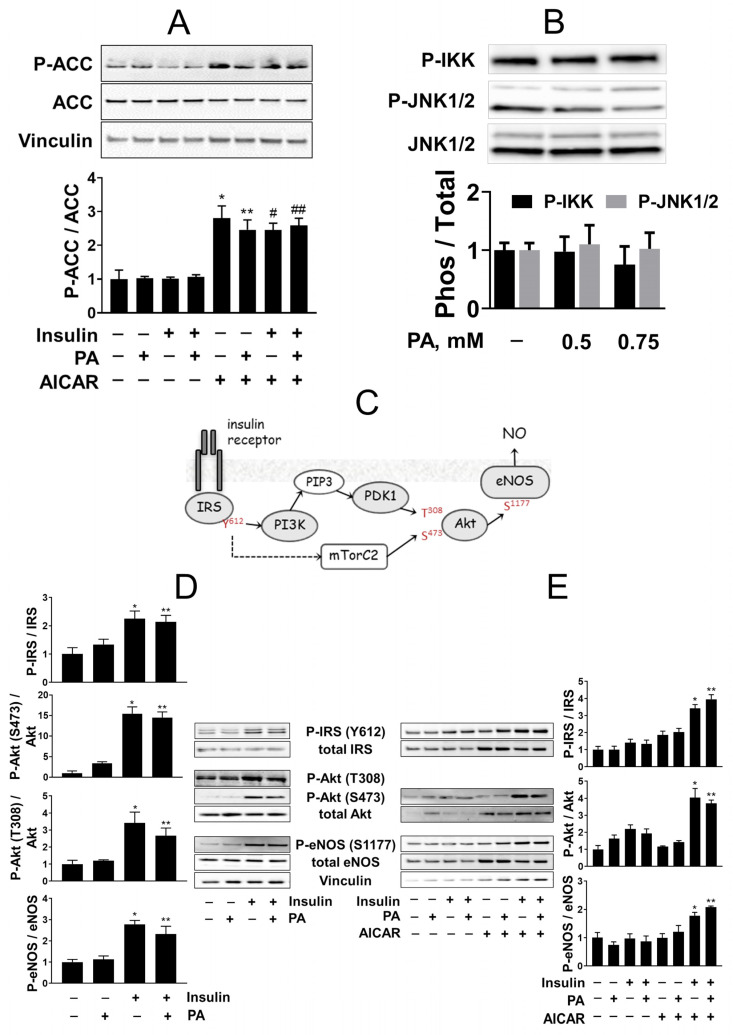
Effects of AICAR and palmitate on AMPK, inflammatory and insulin signaling. Cells were treated with 0.75 mM PA if not indicated otherwise, 100 nM insulin, and 1 mM AICAR. Shown are representative images of at least three independent experiments. Bar graphs represent quantitative analyses of the corresponding Western blots. Data are presented as the mean ± SEM. Mann–Whitney *U*-test was applied to analyze the differences. (**A**) AICAR upregulates, in PA- and insulin-independent manner, phosphorylation of ACC at Ser-79 that reports on AMPK activity. *, *p* < 0.05 vs. untreated control; **, *p* < 0.05 vs. PA; #, *p* < 0.05 vs. insulin; ##, *p* < 0.05 vs. PA + insulin, *n* = 5. (**B**) Activating phosphorylation of inflammatory IKK and JNK1/2 kinases in HUVEC treated with indicated PA concentrations for 5 days, *n* = 2. (**C**) Scheme of insulin signaling in endothelial cells showing the principal components and their phosphorylation sites assessed by Western blots in d–e. (**D**) Insulin signaling in cells treated with 0.75 mM PA for 5 days. *, *p* < 0.05 vs. untreated control; **, *p* < 0.05 vs. PA, *n* = 4. (**E**) Insulin signaling in cells treated with 0.75 mM PA in the presence or absence of 1 mM AICAR for 7 days as indicated. *, *p* < 0.05 vs. untreated control; **, *p* < 0.05 vs. PA, *n* = 3.

To confirm that AMPK activity is stably upregulated in AICAR-treated cells, phosphorylation of acetyl-CoA-carboxylase (P-ACC) at Ser-79 recognized by AMPK was assessed. AICAR similarly increased P-ACC both in the PA-treated and untreated cells irrespective of insulin stimulation (Figure 3A). This indicates that AMPK activity is increased by AICAR in HUVEC, while insulin and palmitate do not interfere. P-ACC levels were also found similarly increased in the AICAR-treated cells at day 7 with no effect of 0.75 mM PA and insulin on P-ACC (data not shown).

### 2.4. Palmitate Does Not Activate Inflammatory IKK and JNK Kinases

Because PA has been shown to induce an inflammatory response in endothelial cells [16,39], potentially leading to apoptosis, we probed the lysates of PA-treated HUVEC with antibodies to the activating phosphorylation sites in the key inflammatory kinases. Neither IKK nor JNK1/2 phosphorylation levels were found substantially increased by PA (Figure 3B).

### 2.5. Insulin Signaling Is Not Directly Impaired by Palmitate, Is Lost in Injured Cells, and Is Protected by AICAR in the Long-Term HUVEC Cultures

To establish the effects of PA and AICAR on insulin signaling in HUVEC, cell lysates were probed by Western blots with antibodies to the key components of insulin signaling to eNOS as its relevant target (Figure 3C). The insulin cascade was responsive to insulin in cells at day 5 of 0.75 mM PA treatment, similar to that in the control (untreated) cells (Figure 3D). However, in cells with reduced viability at day 7 of PA treatment, the insulin cascade was dramatically impaired and failed to respond to insulin (Figure 3E). A somewhat lowered content of total eNOS and vinculin in the lysates of PA-treated cells is consistent with the distorted density of monolayers and reduced cell number when the PA-treated cells lose viability at day 7. In contrast, insulin action was preserved in cells treated by PA even for 7 days in the continuous presence of AICAR and insulin increased phosphorylation of eNOS similarly to that in control cells not treated with PA (Figure 3E). These results suggest that the loss of HUVEC responsiveness to insulin is not a consequence of direct PA action, but rather is contingent on cell injury induced by PA and prevented by AICAR.

### 2.6. AICAR Counteracts Palmitate-Induced NO Production Mediated by eNOS

While PA and AICAR did not affect insulin signaling over the first 5 days, they produced opposing effects on the HUVEC barrier (c.f., Figure 2), possibly acting via NO release. Therefore, we used DAF-FM as a direct NO probe to explore this possibility.

**Figure 4:**
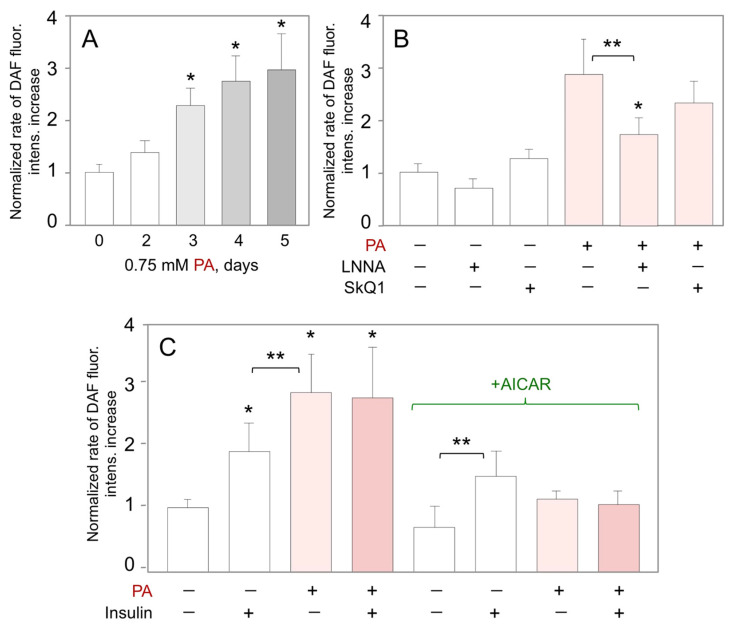
Effects of insulin, palmitate, and AICAR on the rate of NO release by HUVEC. (**A**) Time-dependent changes in the rate of basal NO release in response to 0.75 mM PA (* *p* < 0.05 vs. day 0, *n* = 3). Representative raw fluorescent images of DAF-loaded HUVEC at day 0 and day 5 of treatment with 0.75 mM PA are shown in Supplementary Figure S1. (**B**) Inhibitory analysis of the rate of PA-induced NO release measured at day 5 of cell treatment with 0.75 mM PA (* *p* < 0.05 vs. untreated control; ** *p* < 0.05 as indicated, *n* = 3). (**C**) Effects of AICAR (1 mM, 5 days) on the rate of insulin-stimulated NO release in the presence or absence of 0.75 mM PA for 5 days (*, *p* < 0.05 vs. control cells without AICAR, **, *p* < 0.05 as indicated by brackets, *n* = 3).

In the absence of AICAR, PA time-dependently increased basal (unstimulated) NO production (Figure 4A). This difference was significant after 3 days of cell treatment with PA, which is comparable to the time-frame of the stimulatory PA effect on TER (c.f., Figure 2A). Again, stimulation of basal NO release by PA was dependent on eNOS as it was significantly inhibited by LNNA (Figure 4B). In contrast, the mitochondria-targeted antioxidant SkQ1 [40,41] had a small and insignificant effect on the PA-stimulated NO release (Figure 4B), consistent with cytosolic eNOS localization and no evidence for ROS involvement in NO generation by eNOS. The rate of basal NO production was increased ~3-fold after 5 days of HUVEC treatment with 0.75-mM PA (Figure 4C, left). While insulin increased the rate of NO release ~2-fold in control cells, the stimulation was lost in the PA-treated cells (Figure 4C, left). In the continuous presence of AICAR, both basal, and insulin-stimulated NO release were less but the difference was still ~2-fold (Figure 4C, right). AICAR blocked the PA-induced increase in the rate of basal NO release, but no insulin-stimulated NO release was observed in the presence of PA (Figure 4C, right).

### 2.7. AICAR Counteracts Palmitate-Induced Increase in Mitochondrial ROS

**Figure 5:**
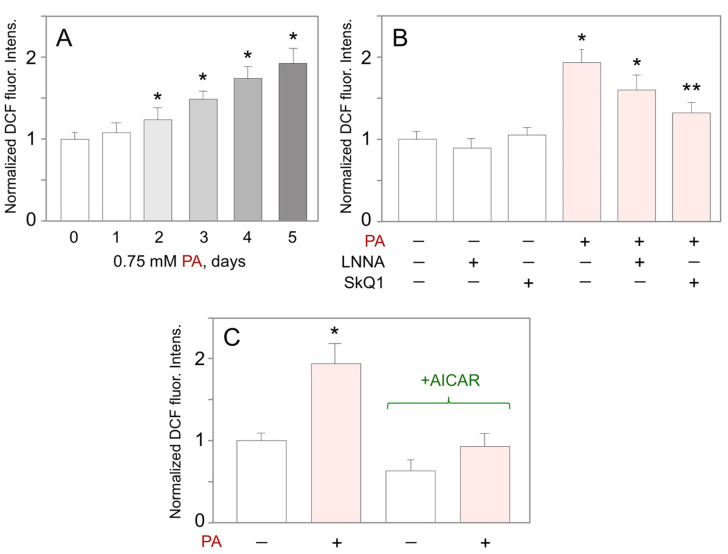
Effects of palmitate and AICAR on ROS production by HUVEC. (**A**) Time-dependent changes in ROS production in response to 0.75 mM PA (* *p* < 0.05 vs. day 0, *n* = 3). (**B**) Inhibitory analysis of PA-induced ROS production measured at day 5 of cell treatment with 0.75 mM PA (* *p* < 0.05 vs. untreated control; ** *p* < 0.05 vs. PA-treated cells without inhibitors, *n* = 3). (**C**) Effects of AICAR (1 mM, 5 days) on ROS production in the presence or absence of 0.75 mM PA for 5 days (* *p* < 0.05 vs. untreated cells without AICAR, *n* = 3).

As mitochondrial FAO may be constantly overburdened by exogenous PA in the presence of carnitine [42], we hypothesized that this may increase mitochondrial ROS. When assessed by DCF-DA assay, 0.75 mM PA steadily built up the intracellular ROS levels in HUVEC (Figure 5A). The mitochondria-targeted ROS quencher, SkQ1, completely blunted the PA-induced accumulation of ROS, whilst LNNA had little effect (Figure 5B). This indicates the mitochondrial origin of the PA-induced ROS. AICAR completely blocked the PA-induced ROS production and returned ROS levels to basal values (Figure 5C).

### 2.8. AICAR Counteracts Palmitate-Induced Protein Labelling by MDA

**Figure 6:**
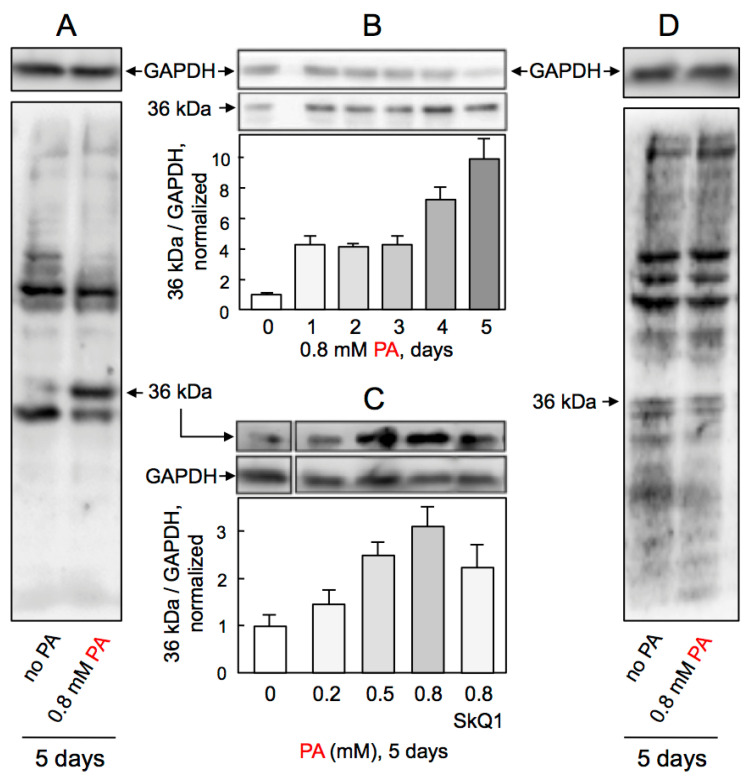
Effects of palmitate and AICAR on protein labeling by MDA in HUVEC. (**A**) Cells were treated by 0.8 mM PA for 5 days and the lysates were probed with antibodies against GAPDH (top) or MDA-labeled proteins (underneath). (**B**) Time-dependent changes in MDA labeling of ~36 kDa protein in response to 0.8 mM PA, *n* = 2. (**C**) Dose-dependent changes in MDA labeling of ~36 kDa protein at day 5 of cell treatment with PA and the effect SkQ1, *n* = 2. (**D**) Cells were treated by 0.8 mM PA for 5 days in continuous presence of 1 mM AICAR; the lysates were probed with antibodies against GAPDH (top) or MDA-labeled proteins (underneath).

ROS are requisite for lipid peroxidation and generation of MDA, the marker of cardiovascular disease and putative mediator of endothelial dysfunction [22]. Using a previously characterized antibody that recognizes the MDA-labeled proteins [23], we explored whether PA increases MDA levels and protein labeling in HUVEC. Western blots of total HUVEC lysates revealed several MDA-labeled protein bands in control cells while exposure to 0.8 mM PA led to a selective increase in the MDA signal associated with a ~36 kDa protein (Figure 6A). This reporter protein signal accumulated in time- (Figure 6B) and dose-dependent manner and was reduced by SkQ1 (Figure 6C). No increased MDA protein labeling was detected in HUVEC treated with 0.8 mM PA in the presence of AICAR (Figure 6D), consistent with the dependence of MDA generation on ROS, which are effectively eliminated by AICAR. Altogether, these results indicate that PA triggers ROS-dependent accumulation of MDA in HUVEC that is attenuated by SkQ1 and prevented by AICAR.

## 3. Discussion

**Figure 7:**
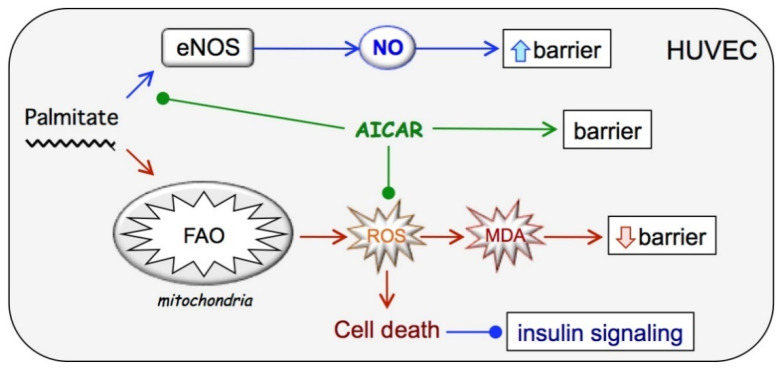
Proposed mechanism of chronic palmitate and AICAR action on endothelial cells. See text for details.

Here, we show that high concentrations of free fatty acid palmitate dramatically reduce the viability of cultured human endothelial cells, confirming the previous observations [12,15,43,44]. PA does not, per se, affect the barrier capacity and insulin signaling in viable cells until they were gradually injured by oxidative stress originating from mitochondria. AICAR effectively negated the PA-induced oxidative stress, vastly increased endothelial cell viability, rescued insulin signaling, and supported long-term barrier maintenance. The obtained results suggest a sequence of events shown schematically in Figure 7.

The relatively narrow range of the blood FFA levels in obese/diabetic vs. healthy individuals has long been established [36,37] and later confirmed for obesity [1,35]. Typically, less than ~0.6 mM FFA could be regarded as a normal level, whereas in obese and T2D individuals it may stably increase to ≥1 mM. Multiple studies that aimed to reveal detrimental effects of FFA on vascular endothelium in vitro used relatively low ambient FFA (0.1–0.5 mM) applied for 48–72 h [11,12,13,14,17,18,19,20,21,25]. They revealed increased apoptosis associated with oxidative stress and inflammatory response, suggesting a poor prognosis for endothelial cells under such conditions. Our HUVEC visualization experiments generally agree with endothelial apoptosis studies and directly demonstrate the detrimental effects of the above-normal PA levels on monolayer integrity. Similarly, the oxidative stress markers associated with PA presence reproduce findings in other studies, including increased MDA levels found after PA treatment of vascular smooth muscle cells [45], or macrophages associated with mitochondrial dysfunction and apoptosis [46]. The initial enhancing effect of PA on the endothelial barrier is novel but its long-term validity is doubtful in the light of soon coming monolayer disintegration due to oxidative injury. Existing discrepancies in timing and PA doses between our and other studies do not belittle the main conclusion that high PA levels are not compatible with endothelial viability in culture. However, endothelial apoptosis is not overwhelming in vivo unless associated with severe pathological states [26,27,28]. Thus, endothelial cell culture conditions need optimization to more closely recapitulate the situation in vasculature.

The seminal studies by Ruderman lab demonstrated that shifting HUVEC metabolism from glycolytic behavior, which is typical for quiescent in vitro cultures, to enhanced FAO by upregulating AMPK in the presence of physiological levels of carnitine [29,30] improves cultured endothelial cell viability [32,34]. Similarly, upregulated FAO and carnitine exerted vasculoprotective effects in animal models [47,48], further suggesting that metabolic state may be critical for endothelial function. This is in line with our findings that HUVEC cultures can be stably maintained in the presence of elevated FFA, such as PA, provided carnitine is present and AMPK is active (Figure 1B). Therefore, these experimental conditions may better suit future obesity- and diabetes-related research of the long-term slowly building effects of dyslipidemia, hyperglycemia, or hyperinsulinemia on vascular endothelium.

In the presence of AICAR, AMPK is activated as judged by increased phosphorylation of its canonical substrate ACC. The stimulatory effect of PA on NO/ROS generation is diminished and cell viability is maintained at a high level. These observations remain in agreement with the antioxidant and antiapoptotic role of AMPK. Increased AMPK activity helps reduce intracellular ROS, possibly via increased UCP-2 expression, the endothelial homolog of UCP-1 (thermogenin), the mitochondrial respiratory chain uncoupler [14,25]. In addition, AMPK phosphorylates ACC to cease malonyl-CoA formation, which is the potent inhibitor of the acyl-carnitine shuttle of fatty acid moieties to mitochondria for oxidation [49]. When AMPK activity is decreased, the long-chain fatty acid acyls can be re-routed to triglyceride and ceramide formation in cytosol increasing the risk of insulin resistance and T2D progression [50]. Endothelial AMPK is activated by shear stress [51], suggesting that in vivo blood flow provides protecting signals to the endothelial lining of the vascular wall to counteract FFA insult. In stationary endothelial culture, AICAR mimics shear stress as an AMPK activator, however direct in vitro assessment of shear stress effects on endothelial tolerance of FFA is important and such studies are underway.

As a proof of concept, we utilized AICAR/carnitine supplementation to address the long-term effects of 0.75 mM PA on endothelial physiology. AICAR prevented the PA-induced NO release by HUVEC but did not alter the expected increase in NO by control cells in response to insulin. However, insulin failed to increase the NO release in the presence of PA, although the insulin signaling towards eNOS was functional in these cells. Currently, no mechanism is available to explain how PA affects NO generation. Palmitoylation of eNOS could be expected, but its contradictory effects on eNOS activity have been noted [52] and need further studies. Regardless, the observed phenomenon of insulin resistance developed in cultured endothelial cells in the presence of PA stays in agreement with the general understanding that NO production by vascular endothelium is decreased in obesity and T2D [4,5,6,7,9].

The ability of AICAR/AMPK to protect endothelium against high PA levels is not endless. Endothelial monolayers maintained in the presence of 1 mM or 1.5 mM PA begin to deteriorate at day 14 or day 8, respectively (Figure 1B). Past this time, the cellular reactions similar to those in standard endothelial culture could be envisioned such as increased ROS generation and MDA modification of endothelial proteins, increased NO generation, as well as contrasting behavior of endothelial barrier. Eventually, these events conclude in oxidative endothelial injury recapitulating more severe conditions of dyslipidemia that are hardly counteracted by endogenous defense mechanisms. Our recent results support this possibility by demonstrating that oxidative stress product MDA readily disturbs actin cytoskeleton, impairs insulin signaling, and disrupts the endothelial barrier [23,24].

In conclusion, vascular endothelial cell culture supplemented with AICAR, an activator of AMPK, and carnitine that facilitates FFA transport in mitochondria for oxidation, provides stable maintenance of human endothelial cells in vitro in the presence of elevated levels of palmitate and allows long-term experiments to address the effects of dyslipidemia on vascular endothelial functions. This in vitro system adequately reproduces insulin resistance in the presence of high levels of palmitate recapitulating insulin resistance in obese and T2D patients. In spite of protection by AMPK, pathologically high levels of palmitate cause oxidative injury and deterioration of endothelial monolayer. Since this injury is mitochondria-related, mitochondria-targeted compound SkQ1 could be considered as a potential novel antioxidant to protect/restore endothelial function in diabetic and cardiovascular patients.

## 4. Materials and Methods

### 4.1. Materials

General reagents, gelatin, and human insulin were purchased from Sigma (USA) and used as before [23,24]. LNNA (N5-[imino(nitroamino)methyl]-L-ornithine) was from Cayman Chemical Company (USA). Defined endothelial basa medium (EBM) and endothelial growth medium (EGM) were from Cell Application (USA). Hank’s balanced salt solution (HBSS), and carnitine were from Lonza (Switzerland). Glutamine, penicillin and streptomycin were from Gibco (USA). Sodium palmitate (Sigma, Burlington, MA, USA) was conjugated with BSA (Amresco, Framingham, MA, USA) as described [53], using 0.2 M palmitate solution in ethanol and 20% BSA in Hank’s solution to obtain the 8 mM stock solution of conjugate, which was subsequently filtered through 30 K Ultra-15 centrifugal filter devices (Amicon, Miami, FL, USA) to remove ethanol (final concentration 0.02%). The conjugate solution was spun at 16,000x *g* for 60 min at 4 °C to remove protein aggregates, if any, and the clarified solution was stored at 4 °C until use. This conjugate is referred to as palmitate in the text below. MDA was obtained by acid hydrolysis of 1,1,4,4-tetrahydroxypropane according to the published protocol [54]. SkQ1 was provided by Mitotech LLC (Moscow, Russia). The Pierce BCA Protein Assay Kit (Thermo Scientific, USA) was used to equalize protein loading for Western blots. For Western blotting, the following antibodies were used: phospho-IRS1 (Tyr612) (# 3203), phospho-Akt (Thr308) (# 9275), phospho-Akt (Ser473) (# 4060), total Akt (# 4691), phospho-IKK (# 2697), (all–Cell Signaling, Loveland, CO, USA), phospho-JNK1/2 (Thr183/Tyr185) (# AF185), total JNK1/2 (# AF1387) (R&D, USA), phospho-eNOS (Ser1177) (# 612392) and total eNOS (# N30020L14) (BD Biosciences, Franklin Lakes, NJ, USA), anti-vinculin (#ab18058, Abcam, Waltham, MA, USA), anti-GAPDH (# MAB374, Chemicon, India), secondary HRP-conjugated goat anti-rabbit (#7074, Cell Signaling, Shirley, NY USA) and rabbit anti-mouse (#A9044, Sigma, Burlington, MA, USA). The antibody against MDA-modified proteins (clone 12E7) was similar to the 6G4 clone used in the previous study [23]. Halt Protease and phosphatase inhibitor cocktail was obtained from ThermoFisher Scientific (Waltham, MA, USA), Clarity ECL from Bio-Rad (Hercules, CA, USA).

### 4.2. HUVEC Treatment by Palmitate and Cell Microscopy

HUVECs of the third passage were cultured in 0.2% gelatin-coated 24-well plates in EGM-2MV growth medium supplemented with 2 mM glutamine, 40 µM carnitine, 100 U/mL penicillin, and 100 μg/mL streptomycin. PA was added to achieve 0.5, 0.75, 1.0, or 1.5-mM final concentration. Where applicable, AICAR was always present at 1 mM final concentration. The media were exchanged daily. Phase-contrast microscopy was performed using an Axio Observer A1 (Zeiss, Germany) inverted microscope equipped with an on-stage thermostat (37 °C) and an AxioCam 506 CCD camera (Zeiss, Oberkochen, Germany). Images were acquired and processed using the AxioVision 4.8.2 software (Zeiss, Germany) and the ImageJ freeware (NIH, Bethesda, MD, USA).

### 4.3. HUVEC Monolayer Permeability

To determine the permeability of HUVEC monolayers, transendothelial electric resistance (TER) assays were performed in the ECIS-z device (Applied Biophysics, Troy, NY, USA). The cells of the third passage were seeded in individual wells of 8W10E electrode arrays (Applied Biophysics, Troy, NY, USA) pre-coated with 0.2–1% gelatin at 1.8 × 105 cells per well in 300 μL of EGM supplemented with 2 mM glutamine, 40 μM carnitine (Lonza, Switzerland), 100 U/mL penicillin and 100 μg/mL streptomycin. The PA-albumin conjugate was added after the cell monolayer had been firmly established to achieve a final palmitate concentration of 0.75 mM (assigned as day 0). LNNA was added to 0.3 mM in some experiments along with PA. The cells were further cultured for up to 11 days with continuous monitoring of TER using an ECIS-z system (Applied Biophysics, Troy, NY, USA). The medium was exchanged on the daily basis keeping the FFA and LNNA concentrations constant.

### 4.4. NO Accumulation Assay

HUVEC of the third passage were seeded into 96-well cell culture microplates (μclear black, Cellstar, TC, F-bottom, Greiner Bio-One, Kremsmünster, Austria) coated with 0.2% gelatin at 0.5 × 10^5^ cells per well in 100 μL of EGM supplemented with 2 mM glutamine, 40 μM carnitine, 100 U/mL penicillin and 100 μg/mL streptomycin. After reaching confluency, PA and/or LNNA were added, respectively, to 0.75 mM or 0.3 mM followed by daily medium exchange keeping PA and LNNA concentrations constant. SkQ1 was added once to 2 μM 24 h prior to PA. Then, the medium was exchanged for 1 h for EBM and then for 100 μL EBM containing 2 μM DAF-FM diacetate, 1 mM probenecid (both–Invitrogen, Carlsbad, CA, USA), and 15 mM HEPES for 30 min. Then the medium was exchanged to 100 μL EBM containing only 1 mM probenecid and 15 mM HEPES. DAF fluorescence was registered using an AxioVert 200 M microscope equipped with a High-Speed AxioCam HSm cooled CCD camera working in full chip mode. Insulin (Novo Nordisk, Denmark) was added to appropriate wells to 100 nM final concentration. DAF fluorescence signals were acquired from 200–400 cells using the ×10 objective. Images were taken once in 10 min for 40–60 min with minimum expose intensity and 0.8 s time to minimize any photobleaching effects. Following NO measurement under the microscope, the integral fluorescence intensity of HUVEC acquired during the DAF loading step due to constitutive eNOS activity (image 0, F_0_) was digitally subtracted from the integral fluorescence intensity of every subsequent image (image *n*, F_n_-F_0_). Adjusted DAF fluorescence intensity values were normalized by the number of DAF-positive cells in each image and plotted as the time course of NO accumulation per cell. From the linear part of the NO accumulation curve, the rate of NO release was calculated as a tangent value of DAF fluorescence intensity increase over 10 min. The rate of NO release in a particular experiment was normalized by the rate of NO release in the corresponding control and plotted as a bar graph. A representative movie of DAF fluorescence intensity increase in HUVEC used for calculation of the rate of NO release is available in the Supplementary Materials (file DAF_HUVEC time lapse. avi).

### 4.5. ROS Accumulation Assay

HUVECs of the third passage were cultured in black F-bottom 96-well microplates as above for 5 days after PA addition to 0.75 mM. LNNA or SkQ1 were added as above. Then, the medium was exchanged daily. Prior to ROS measurements, it was changed to EBM for 1 h, then to EBM containing 10 μM DCF-DA (Sigma, Burlington, MA, USA) for 60 min, to EBM for 30 min, and finally to Hank’s solution containing 15 mM HEPES for 30 min, after which DCF fluorescence was measured in a Victor X3 plate reader (Perkin Elmer, Waltham, MA, USA). Background fluorescence was determined in parallel in cells not treated with DCF-DA; these values were subtracted from those of fluorescence of DCF-DA-treated cells. The data were expressed as the difference between DCF fluorescence of the cells treated with PA, SkQ1, or LNNA and the control untreated cells, normalized to that in the control cells.

### 4.6. Western Blotting

HUVECs were grown up to confluency in 6-well plates coated with 0.2% gelatin in EGM containing 2 mM glutamine, 40 µM carnitine, 100 U/mL penicillin, and 100 μg/mL streptomycin. Cells were treated with 0.75 mM PA or with media containing 0.75 mM PA and 1 mM AICAR for desired time with the daily medium exchange. Cells were deprived for 2 h in EBM and stimulated by 100 nM insulin for 20 min. Then, the cells were washed twice with an ice-cold PBS and lysed in 2-fold Laemmli sample buffer containing the Halt Protease and Phosphatase inhibitor cocktail. The lysates were passed 10 times through a 30-gauge syringe needle to disrupt DNA, heated to 56 °C for 30 min, and clarified by centrifugation at 16,000 g at 4 °C for 10 min. Western blotting was performed using Criterion devices (Bio-Rad, Hercules, CA, USA) at 0.4 A*hour for protein transfer. The protein bands were detected by Clarity ECL reagents (Bio-Rad, Hercules, CA, USA) and visualized by a Fusion-SL 3500WL instrument (Vilber Lourmat, Collégien, France).

### 4.7. Statistics

The data were analyzed using Student’s *t*-test and presented as mean ± SD. Experiments were carried out in triplicates or quadruplicates each and repeated *n* times as indicated in the figure legends. For Western blot quantitative analyses, Mann–Whitney *U*-test was used.

## Data Availability

The datasets analyzed during the current study are available from the corresponding author upon reasonable request.

## Supplementary Materials

The following are available online at https://www.mdpi.com/article/10.3390/ijms23010211/s1.
