# Supplementary figures and images for: AICAR Protects Vascular Endothelial Cells from Oxidative Injury Induced by the Long-Term Palmitate Excess
Source: Int J Mol Sci. 2021 Dec 25;23(1):211. doi: 10.3390/ijms23010211 (PMC8745318; doi:10.3390/ijms23010211)

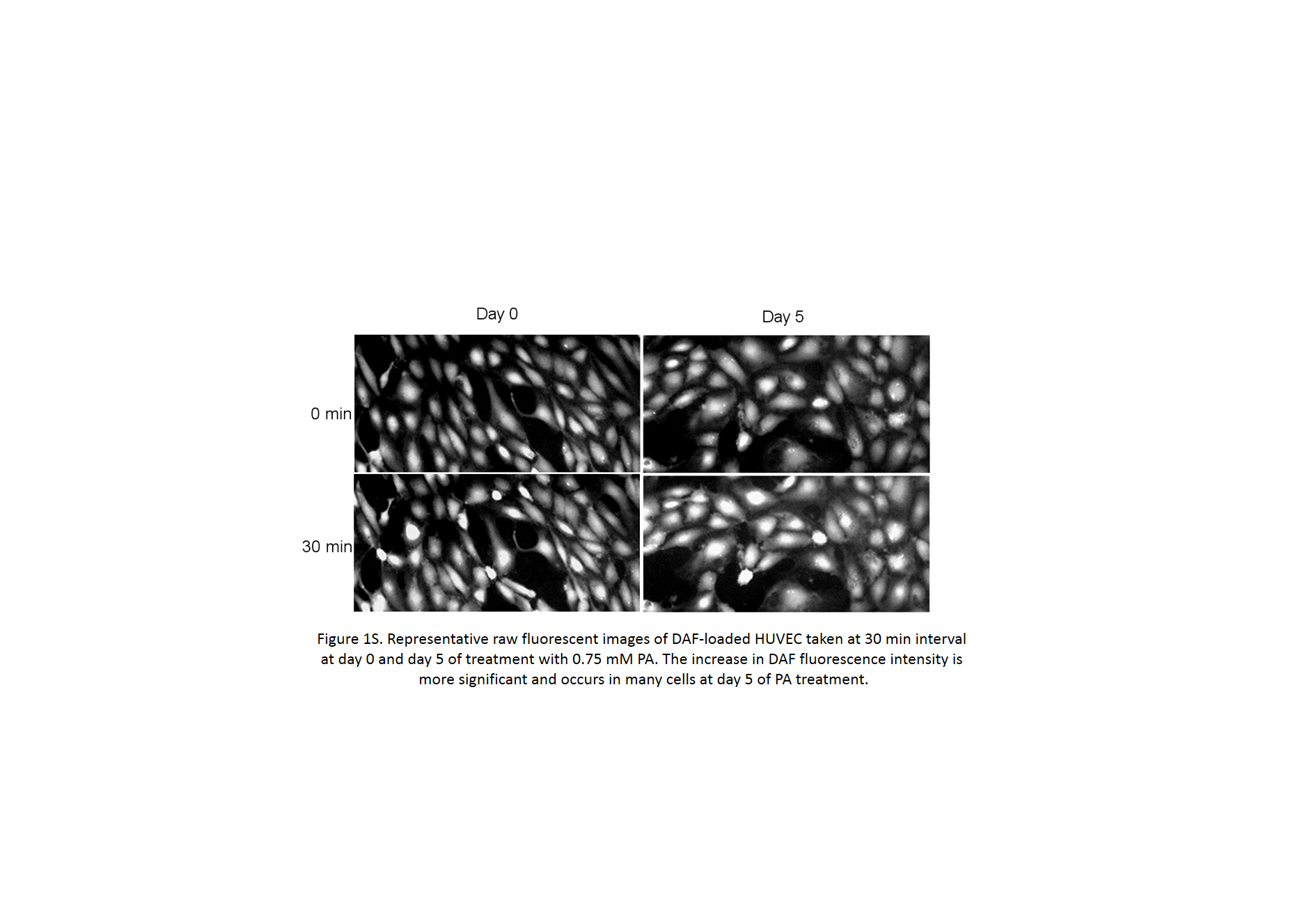

Supplement: Supplementary file 1 [file ijms-23-00211-s001.zip › Supplementary files/fig.1S.tif]

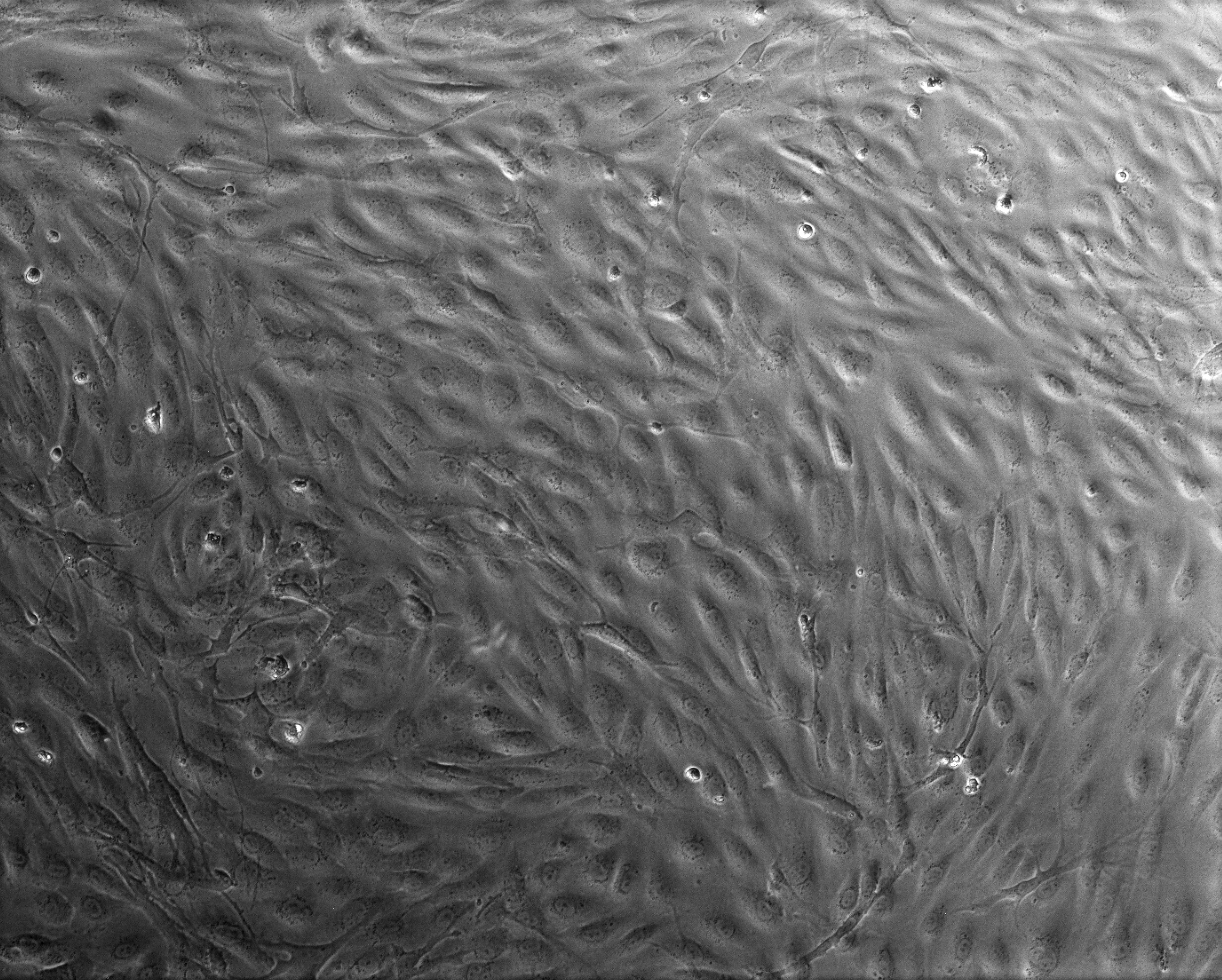

Supplement: Supplementary file 1 [file ijms-23-00211-s001.zip › Supplementary files/HUVEC 0.75 mM PA 1 mM AICAR days 4 6 8 10 14/HUVEC 0.75 mM PA 1 mM AICAR day10.tif]

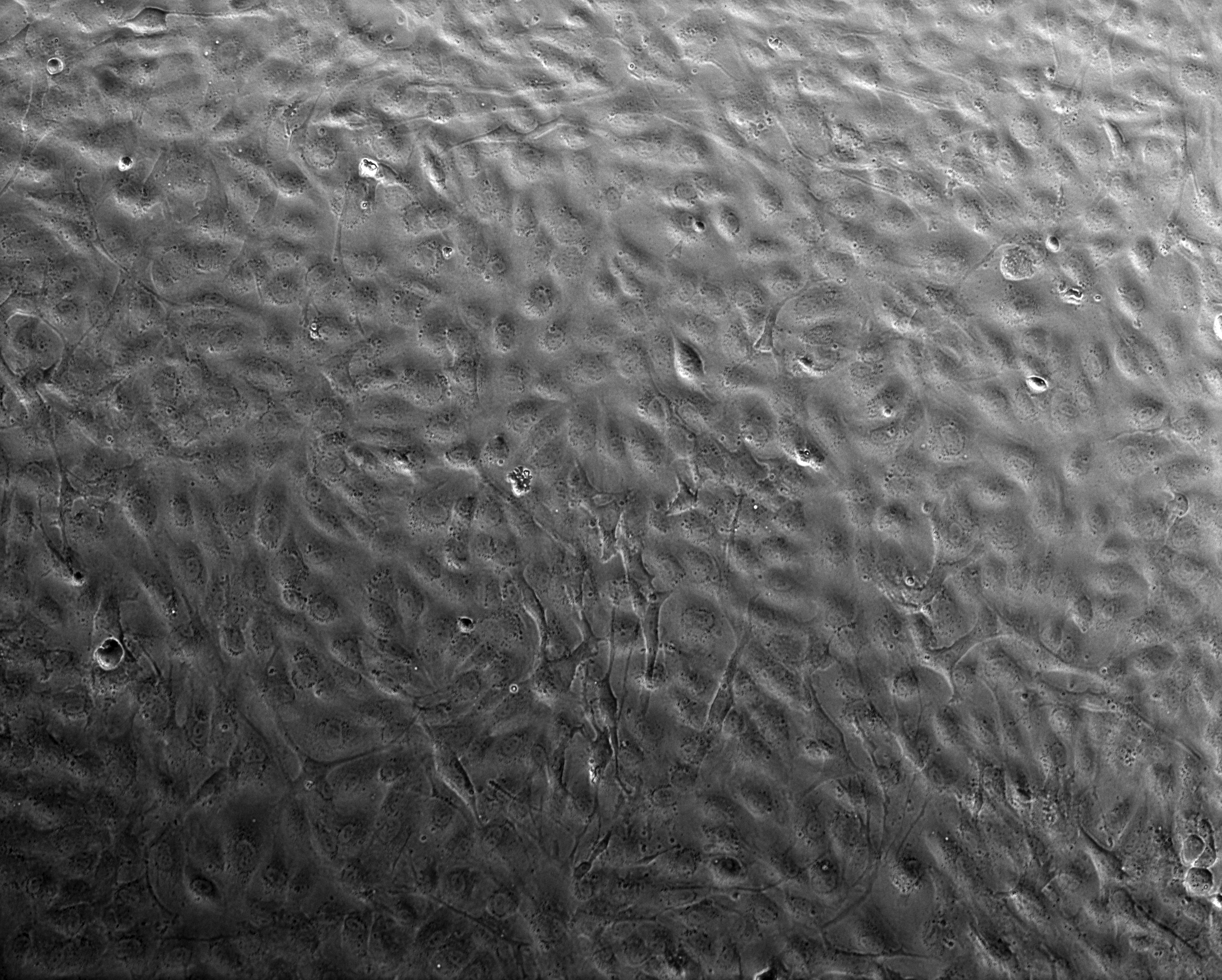

Supplement: Supplementary file 1 [file ijms-23-00211-s001.zip › Supplementary files/HUVEC 0.75 mM PA 1 mM AICAR days 4 6 8 10 14/HUVEC 0.75 mM PA 1 mM AICAR day14.tif]

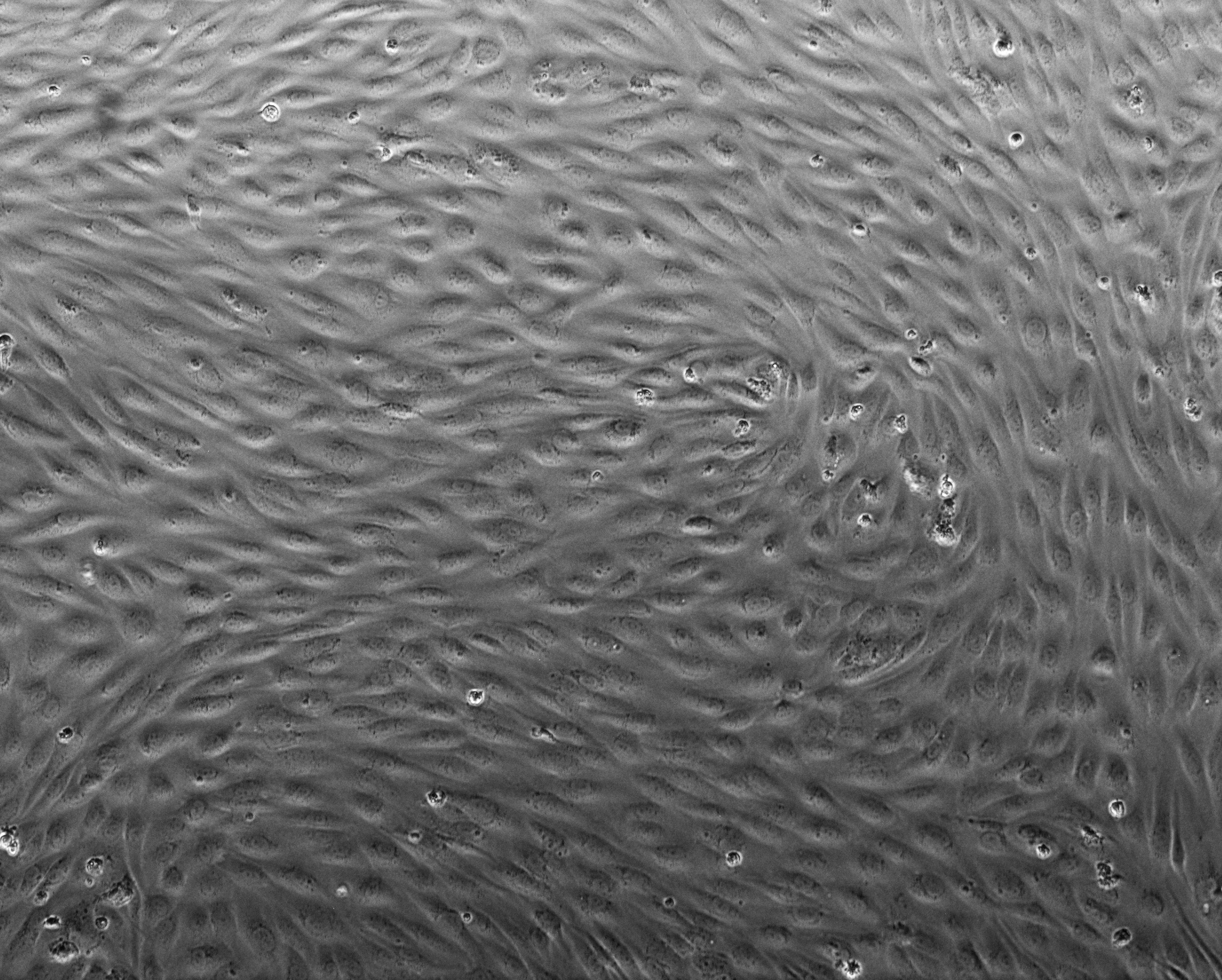

Supplement: Supplementary file 1 [file ijms-23-00211-s001.zip › Supplementary files/HUVEC 0.75 mM PA 1 mM AICAR days 4 6 8 10 14/HUVEC 0.75 mM PA 1 mM AICAR day4.tif]

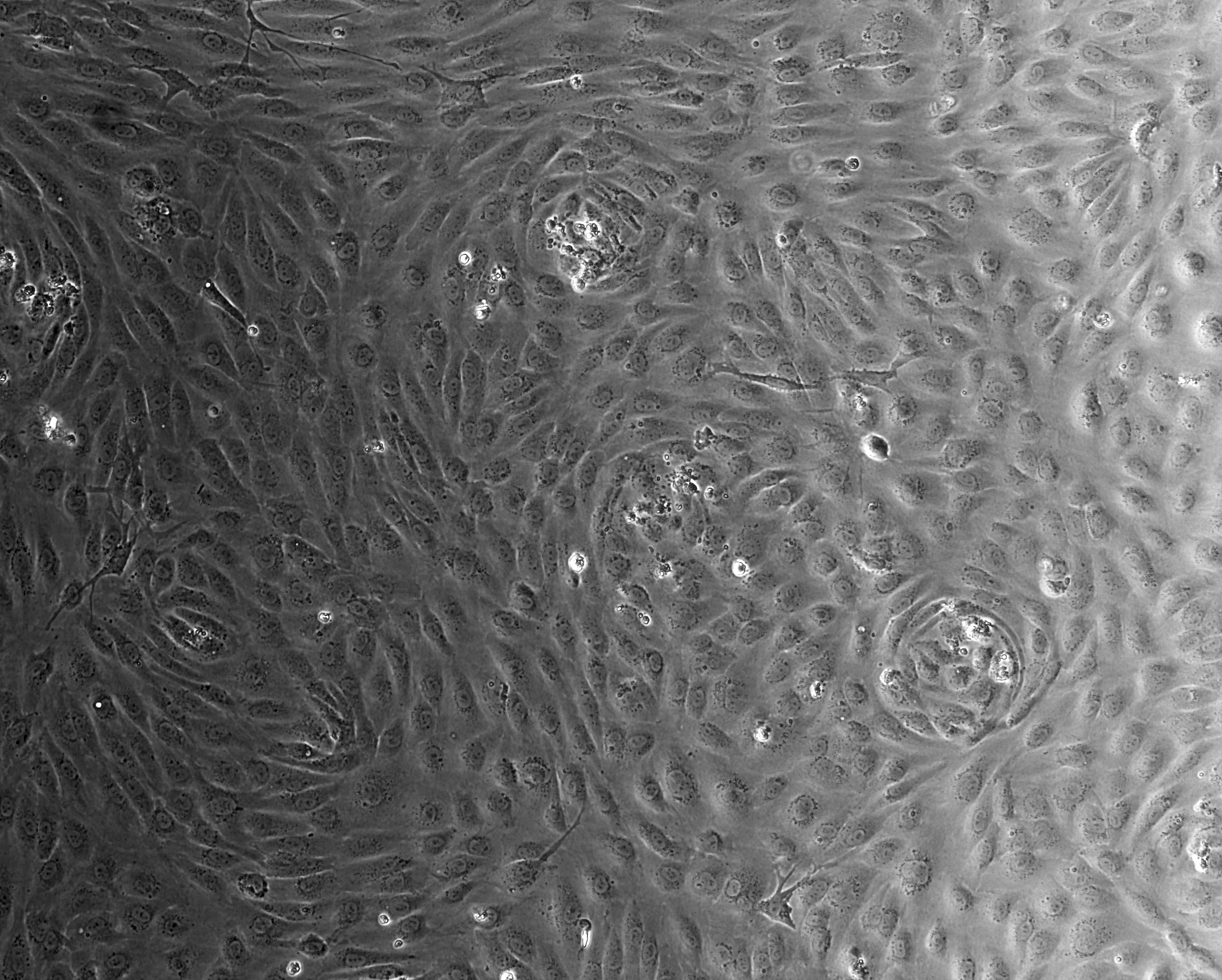

Supplement: Supplementary file 1 [file ijms-23-00211-s001.zip › Supplementary files/HUVEC 0.75 mM PA 1 mM AICAR days 4 6 8 10 14/HUVEC 0.75 mM PA 1 mM AICAR day6.tif]

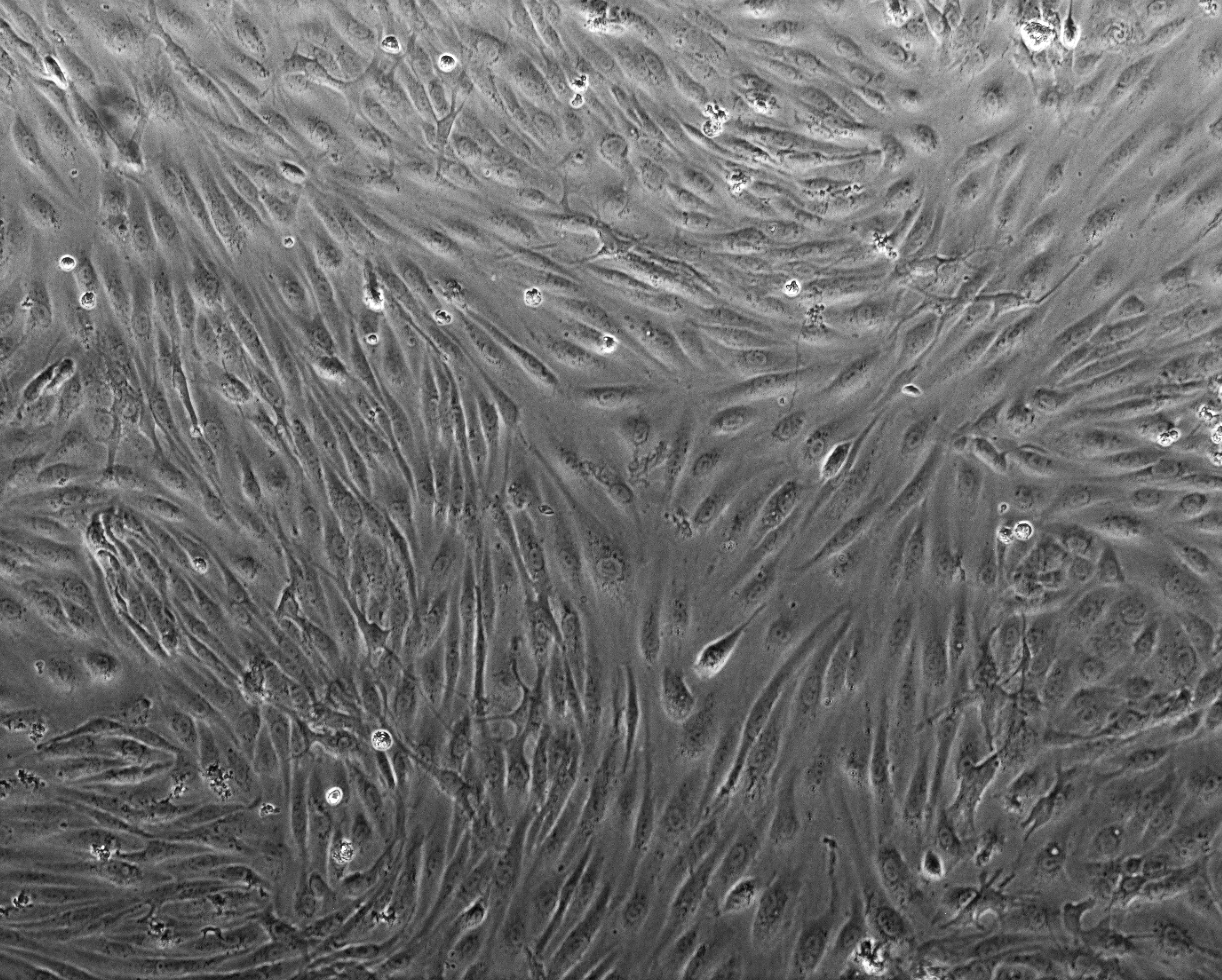

Supplement: Supplementary file 1 [file ijms-23-00211-s001.zip › Supplementary files/HUVEC 0.75 mM PA 1 mM AICAR days 4 6 8 10 14/HUVEC 0.75 mM PA 1 mM AICAR day8.tif]

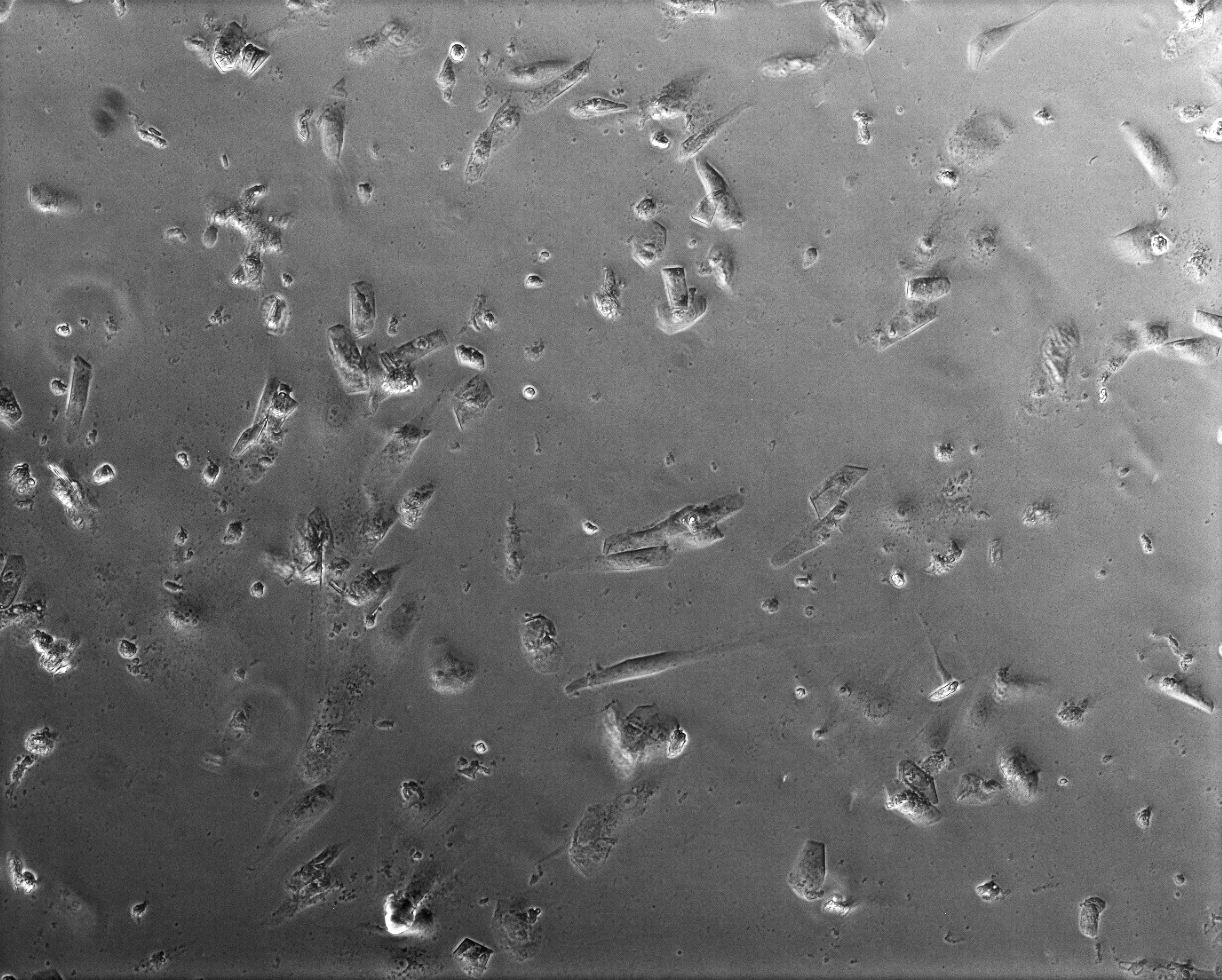

Supplement: Supplementary file 1 [file ijms-23-00211-s001.zip › Supplementary files/HUVEC 0.75 mM PA days 4 6 8 10 14/HUVEC 0.75 mM PA day10.tif]

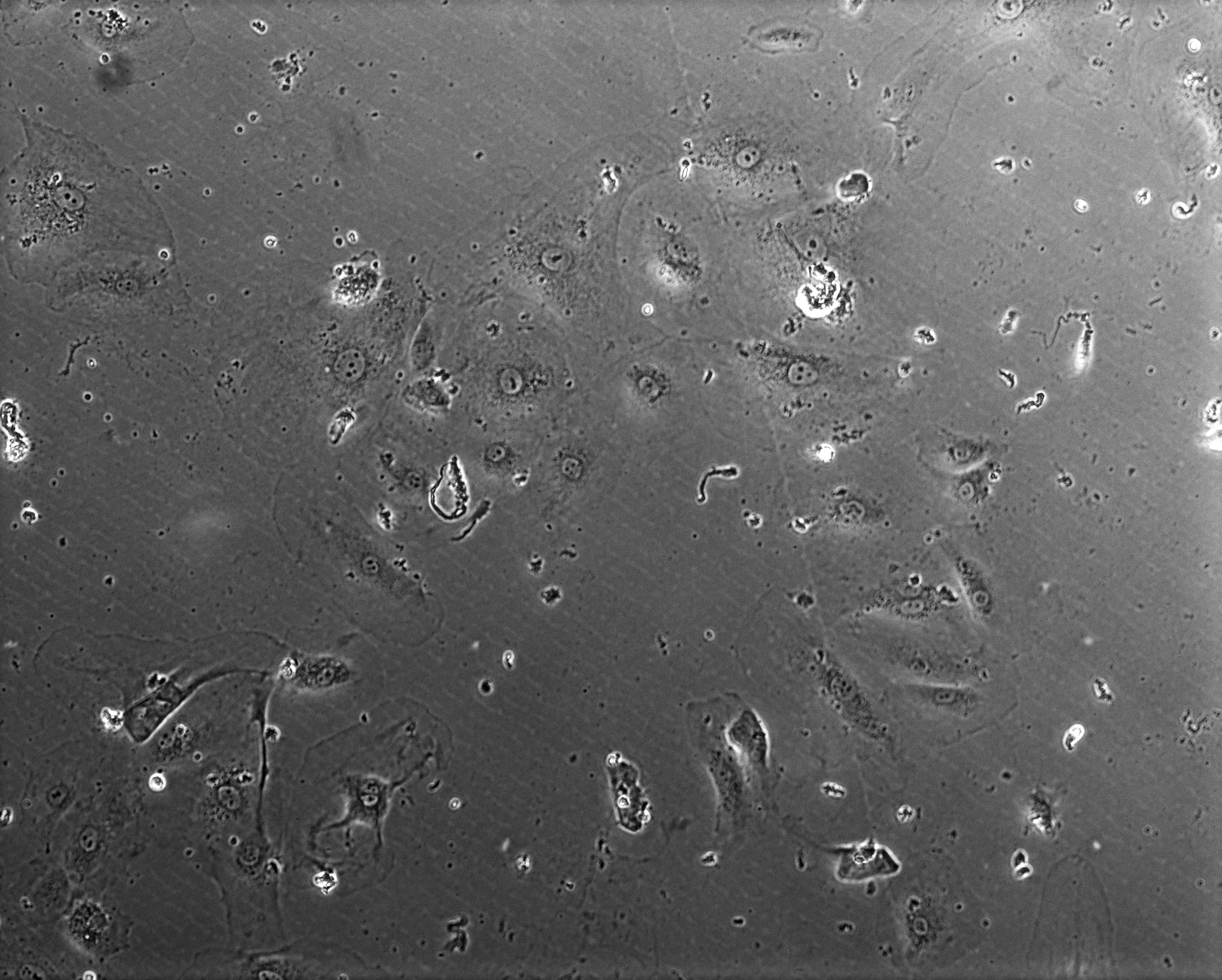

Supplement: Supplementary file 1 [file ijms-23-00211-s001.zip › Supplementary files/HUVEC 0.75 mM PA days 4 6 8 10 14/HUVEC 0.75 mM PA day14.tif]

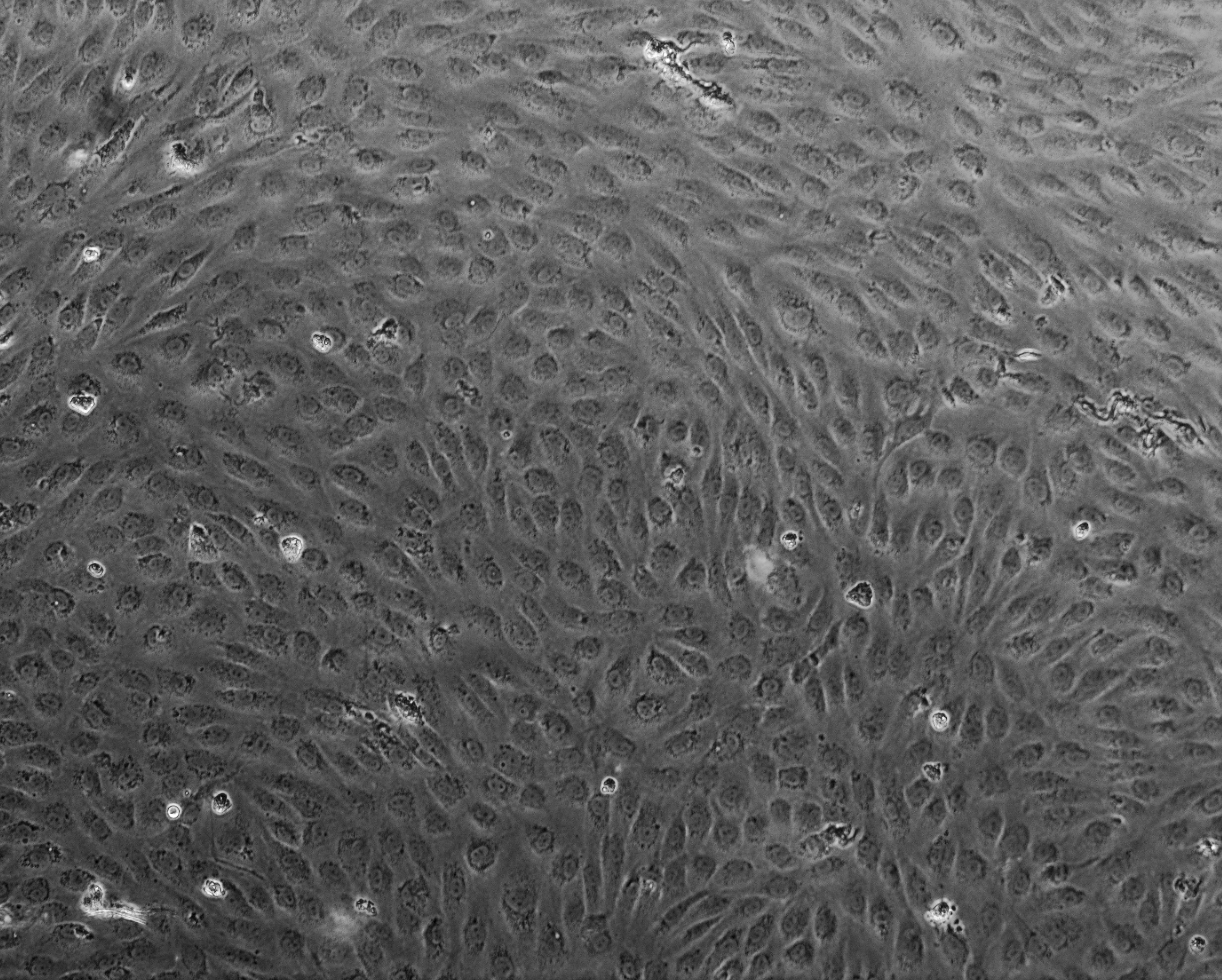

Supplement: Supplementary file 1 [file ijms-23-00211-s001.zip › Supplementary files/HUVEC 0.75 mM PA days 4 6 8 10 14/HUVEC 0.75 mM PA day4.tif]

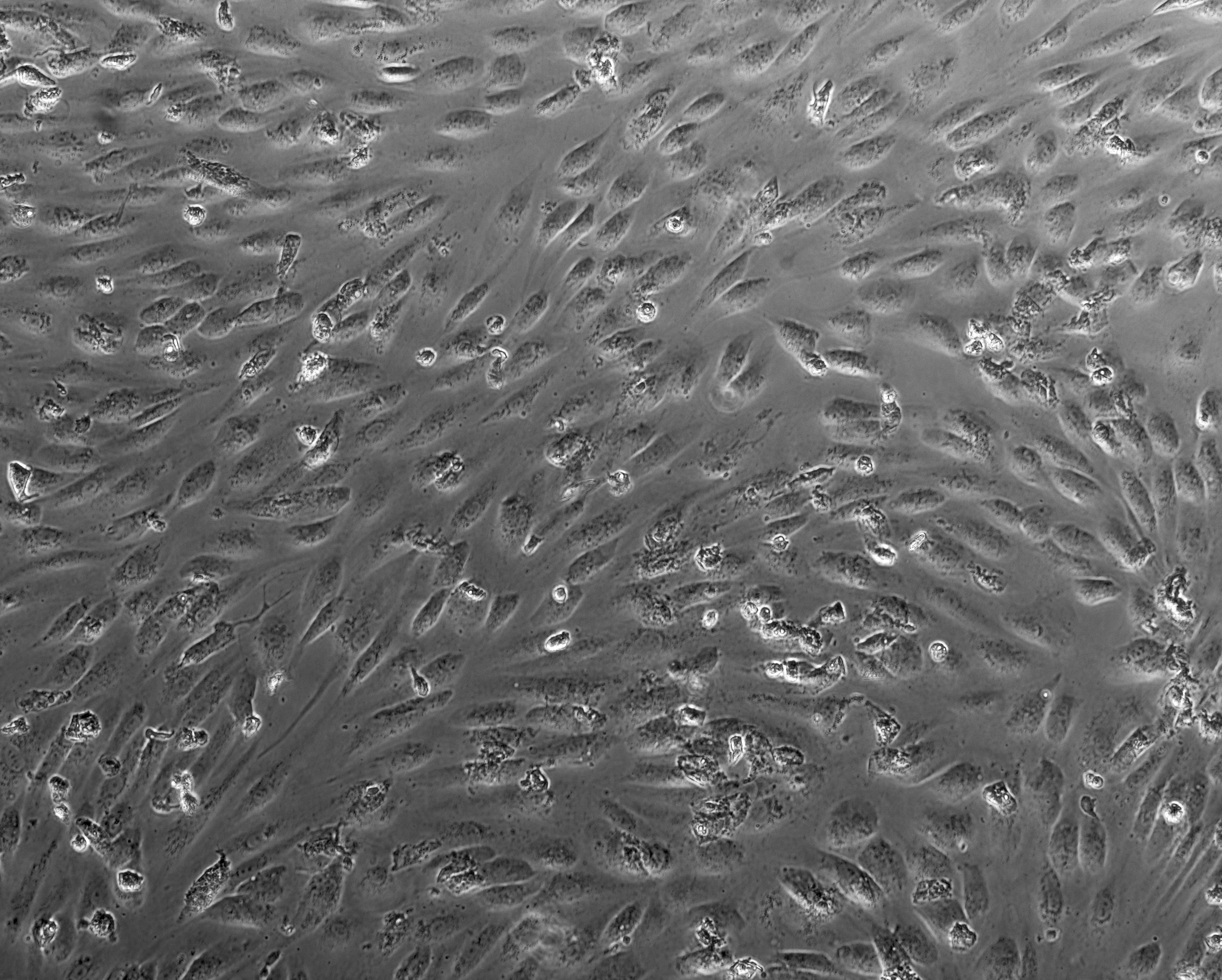

Supplement: Supplementary file 1 [file ijms-23-00211-s001.zip › Supplementary files/HUVEC 0.75 mM PA days 4 6 8 10 14/HUVEC 0.75 mM PA day6.tif]

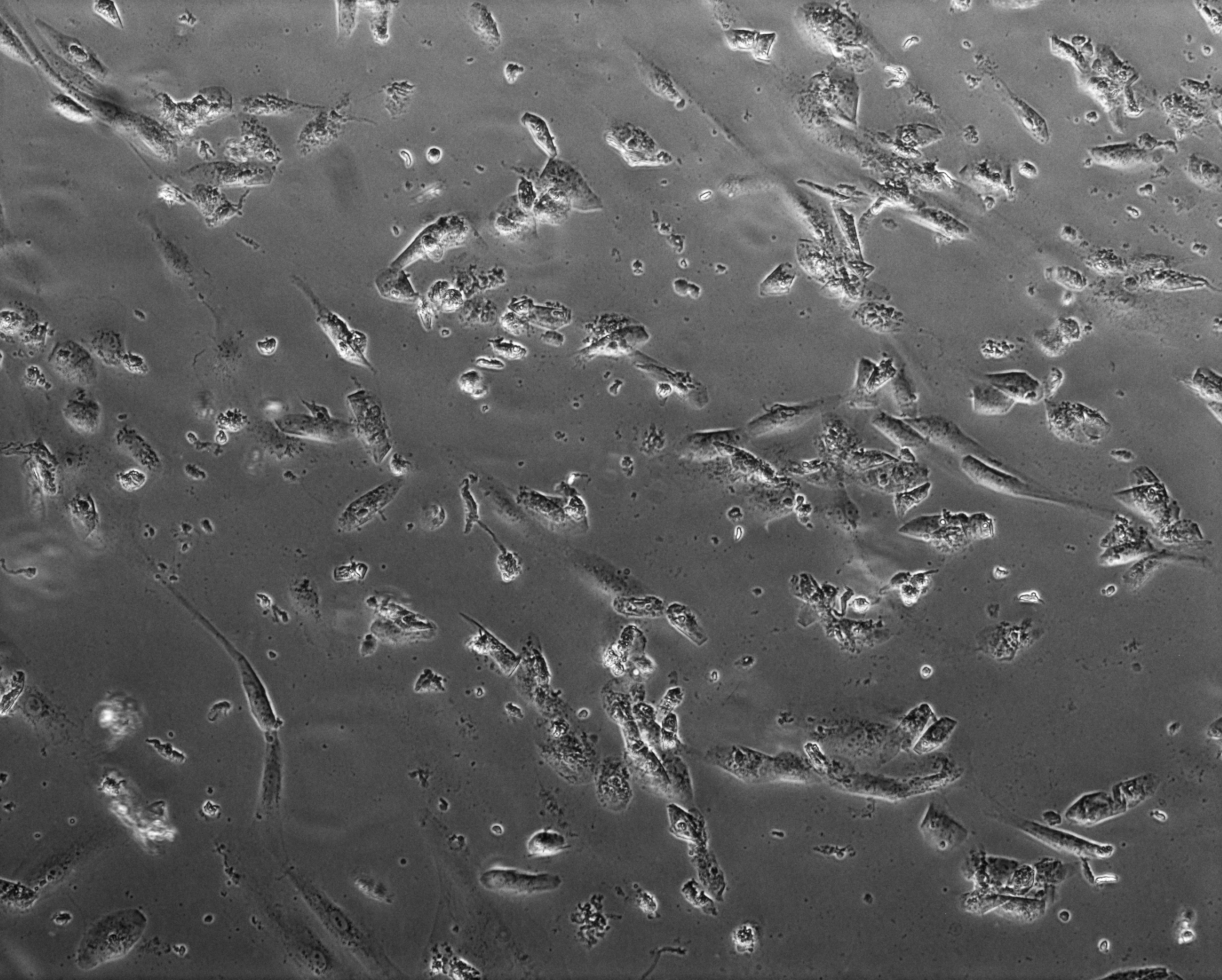

Supplement: Supplementary file 1 [file ijms-23-00211-s001.zip › Supplementary files/HUVEC 0.75 mM PA days 4 6 8 10 14/HUVEC 0.75 mM PA day8.tif]
